# Construction of Vascular Tissues with Macro-Porous Nano-Fibrous Scaffolds and Smooth Muscle Cells Enriched from Differentiated Embryonic Stem Cells

**DOI:** 10.1371/journal.pone.0035580

**Published:** 2012-04-24

**Authors:** Jiang Hu, Changqing Xie, Haiyun Ma, Bo Yang, Peter X. Ma, Y. Eugene Chen

**Affiliations:** 1 Department of Biologic and Materials Sciences, University of Michigan, Ann Arbor, Michigan, United States of America; 2 Department of Internal Medicine, Cardiovascular Center, University of Michigan, Ann Arbor, Michigan, United States of America; 3 Department of Cardiac Surgery, Cardiovascular Center, University of Michigan, Ann Arbor, Michigan, United States of America; 4 Department of Biomedical Engineering, University of Michigan, Ann Arbor, Michigan, United States of America; 5 Macromolecular Science and Engineering Center, University of Michigan, Ann Arbor, Michigan, United States of America; 6 Department of Materials Science and Engineering, University of Michigan, Ann Arbor, Michigan, United States of America; William Harvey Research Institute, Barts and The London School of Medicine and Dentistry, Queen Mary University of London, United Kingdom

## Abstract

Vascular smooth muscle cells (SMCs) have been broadly used for constructing tissue-engineered blood vessels. However, the availability of mature SMCs from donors or patients is very limited. Derivation of SMCs by differentiating embryonic stem cells (ESCs) has been reported, but not widely utilized in vascular tissue engineering due to low induction efficiency and, hence, low SMC purity. To address these problems, SMCs were enriched from retinoic acid induced mouse ESCs with *LacZ* genetic labeling under the control of SM22α promoter as the positive sorting marker in the present study. The sorted SMCs were characterized and then cultured on three-dimensional macro-porous nano-fibrous scaffolds *in vitro* or implanted subcutaneously into nude mice after being seeded on the scaffolds. Our data showed that the *LacZ* staining, which reflected the corresponding SMC marker SM22α expression level, was efficient as a positive selection marker to dramatically enrich SMCs and eliminate other cell types. After the sorted cells were seeded into the three-dimensional nano-fibrous scaffolds, continuous retinoic acid treatment further enhanced the SMC marker gene expression level while inhibited pluripotent maker gene expression level during the *in vitro* culture. Meanwhile, after being implanted subcutaneously into nude mice, the implanted cells maintained the positive *LacZ* staining within the constructs and no teratoma formation was observed. In conclusion, our results demonstrated the potential of SMCs derived from ESCs as a promising cell source for therapeutic vascular tissue engineering and disease model applications.

## Introduction

Vascular smooth muscle cells (SMCs) constitute the principal layer of all small and large blood vessels in the body. Functional deficiency of vascular SMCs plays a central role in the progression of a large number of major human diseases [1], [2]. Many therapeutic treatments to cardiovascular diseases have either enhanced *in situ* blood vessel formation or implanted *in vitro* constructed blood vessels. The efforts to construct tissue-engineered blood vessels *in vitro* using mature vascular SMCs isolated from donor vascular tissues have been extensively reported [3]–[5]. However, mature vascular SMCs have limited ability to proliferate and the cell source is very limited. Embryonic stem cells (ESCs) have shown promise as a therapeutic cell source because of the capacity of self-renewal and the potential to differentiate into various cell types in the adult body. A growing number of studies have demonstrated the differentiation capabilities of ESCs into lineage-specific cells under the appropriate stimuli and the control of a range of signal pathway complex [6]. We and others have utilized a two-dimensional monolayer culture system for cardiovascular differentiation [7]–[9], which has facilitated the dissection of molecular and cellular mechanisms of cardiovascular development [10], [11]. However, one of the major challenges for the application of ESC-derived SMCs to the construction of functional vascular tissues is the heterogeneous cell population derived from pluripotent stem cells, which may lead to teratoma formation or inferior tissue organization. Thus strategies have to be developed to efficiently induce and enrich SMCs before the cells can be used for vascular tissue engineering. Another challenge is to develop a suitable porous scaffold to support vascular tissue development. We recently developed three-dimensional (3D) macro-porous nano-fibrous (NF) scaffolds and found them to preferentially support contractile phenotype of human aorta SMCs under *in vitro* culture conditions [3], which may therefore serve as a suitable scaffold for constructing vascular tissues. In this study, SMCs derived from the differentiated mouse SM22α^−/−*LacZ*^ ESCs were enriched by positive cell sorting, and the 3D NF scaffolds were shown to support the growth of sorted cells both *in vitro* and *in vivo.*

## Materials and Methods

### SMC differentiation of SM22α^−/−*LacZ*^ ESCs

SM22α^−/−*LacZ*^ ESCs were kindly provided by Dr. Li Li from Wayne State University, who published the SM22α-KO mice data previously [12]. The ESCs were expanded and induced to differentiate into SMCs *in vitro* when treated with 10^−5^ M all trans-retinoid acid (RA) following the protocol described in our previous publication [8]. The vehicle dimethyl sulfoxide (DMSO) treatment served as a spontaneous differentiation control.

### β-gal staining

A β-gal staining kit (Invitrogen, Carlsbad, CA) was used for β-gal staining. The derived cells after treatment with either the DMSO or RA for different time periods were fixed with 2% formaldehyde and incubated with an X-gal containing solution, according to the manufacturer's instructions. For β-gal staining of the implants, sections (5 µm thick) were generated using a cryostat (CM 1850, Leica Microsystems) and thaw-mounted onto glass slides and stored at −20°C until staining. The cell nuclei were counterstained with fast red (Vector Lab, Burlingame, CA). Implant samples were also stained with hematoxylin and eosin (H&E).

### Fluorescence activated cell sorting (FACS)

Cell suspension preparation and FACS were performed following published methods [13]. Briefly, differentiated cells cultured on Petri dishes were digested with trypsin and then centrifuged to remove the supernatant. Cells were re-suspended in staining medium to a density of ∼10^7^ cells/mL and loaded with 5-chloromethylfluorescein di-β-D-galactopyranoside (FDG) by adding 100 µL 2 mM pre-warmed FDG working solution. Cells were mixed rapidly and incubated in a 37°C water bath for 1 minute. Then the FDG loading was stopped by adding 1.8 mL ice-cold staining buffer. The cells were kept on ice prior to FACS. The background autofluorescence compensation was set with unstained cells. The cells with fluorescence labeling (*LacZ* positive) were analyzed and sorted using the FACSCalibur™ system (BD Biosciences, San Jose, CA) following the user's guide.

### Immunofluorescence staining

Sorted cells were replated on 0.1% gelatin-coated chamber vessels (BD Biosciences) and incubated in the culture medium overnight. Cells were then fixed, permeabilized and incubated overnight with smooth muscle-α-actin (α-SMA) antibody at 1∶1000 dilutions (Millipore, Temecula, CA). After rinsing, samples were incubated with Alexa Fluor-488 conjugated secondary antibody (Molecular Probe, Eugene, OR) for 1 hour. The cell nuclei were co-stained with 4′,6-diamidino-2-phenylindole (DAPI) (Molecular Probe). Samples were observed under an Olympus BX51 microscope.

### Fabrication of 3D macro-porous NF scaffolds

3D macro-porous NF scaffolds were fabricated following our previous publication [3]. Briefly, 10wt/v% poly(l-lactide) (PLLA) (Boehringer Ingelheim, Ingelheim, Germany) in tetrahydrofuran solution was cast into an assembled sugar template from bound sugar spheres with a diameter of 125–250 µm under a mild vacuum. After phase separation at −20°C overnight, the polymer-sugar composite was immersed into cyclohexane to exchange tetrahydrofuran for 2 days. The resulting polymer-sugar composites were freeze-dried and the sugar spheres were leached out in distilled water. The obtained macro-porous NF scaffolds were freeze-dried again and cut into circular disks with dimensions of 3.6 mm in diameter and 1 mm in thickness. The scaffolds were sterilized with ethylene oxide before use.

### Cell culture on 3D macro-porous NF scaffolds

The sterile 3D NF scaffolds were pre-wetted in 70% ethanol for 30 minutes, washed three times with PBS for 30 minutes each, and twice in cell culture medium for 2 hours each. 2.5×10^5^ cells were seeded on each scaffold. The cell-seeded scaffolds were then transferred to 6-well plates on an orbital shaker [14]. The medium was changed twice a week.

### Scanning electron microscopy (SEM) observation

Samples were rinsed in PBS, fixed in 2.5% glutaraldehyde and 2% paraformaldehyde overnight, and post-fixed in 1% osmium tetroxide for 1 hour. Samples were dehydrated in increasing concentrations of ethanol and hexamethyldisilizane. The samples were then sputter-coated with gold and observed under a scanning electron microscope (Philips XL30 FEG).

### Gene expression analysis with quantitative real-time PCR

Total cellular RNA and cDNA were prepared with an RNeasy mini kit (Qiagen, Valencia, CA) and superscript III first-strand synthesis method (Invitrogen, Carlsbad, CA) following the manufacturer's instructions respectively. PCR amplification was performed with primers selective for individual genes with SYBR Green supermix kit (Bio-rad, Hercules, CA). PCR primers and reaction conditions were described previously [11] and in Table S1. All gene expression level of individual RNA sample was normalized against 18S RNA as an internal standard.

### Histological observation

The cell-scaffold constructs were washed in PBS, fixed with 3.7% formaldehyde, dehydrated in ethanol, and embedded in paraffin. Sections (5 µm thick) were stained with H&E method. For immunofluorescence analysis of SMC specific markers, the slides were de-paraffinized, blocked with serum, incubated with primary antibodies to α-SMA (1∶50 dilution, Millipore) and then incubated with Alexa Fluor-592 conjugated secondary antibodies (Invitrogen, Carlsbad, CA). Samples were observed under an Olympus BX51 microscope.

### Subcutaneous implantation

After the sorted SMCs were seeded and cultured on scaffolds for 24 hours, the cell-scaffold constructs were implanted into subcutaneous pockets on nude mice. Briefly, 6–8 weeks old male nude mice (Charles River Laboratories, Wilmington, MA) were anesthetized with isofluorane. Two dorsal midsagittal incisions were made on the disinfected back. One subcutaneous pocket was created on each side of each incision using blunt dissection and one scaffold was implanted into each pocket. Four samples were implanted randomly for each group (n = 4). The mice were euthanized and the implants were harvested at 2 weeks for β-gal staining. The animal procedures followed the approved protocol by the University of Michigan Committee on Use and Care of Laboratory Animals.

### Statistical analysis

Numerical data were reported as mean ± S.D. from triplicate cell culture (n = 3). The Student's *t*-test was applied to test the significance between the groups. The value of *p*<0.05 was considered statistically significant.

## Results

### Enrichment of SMCs from differentiated SM22α^−/−*LacZ*^ ESCs

**Figure 1:**
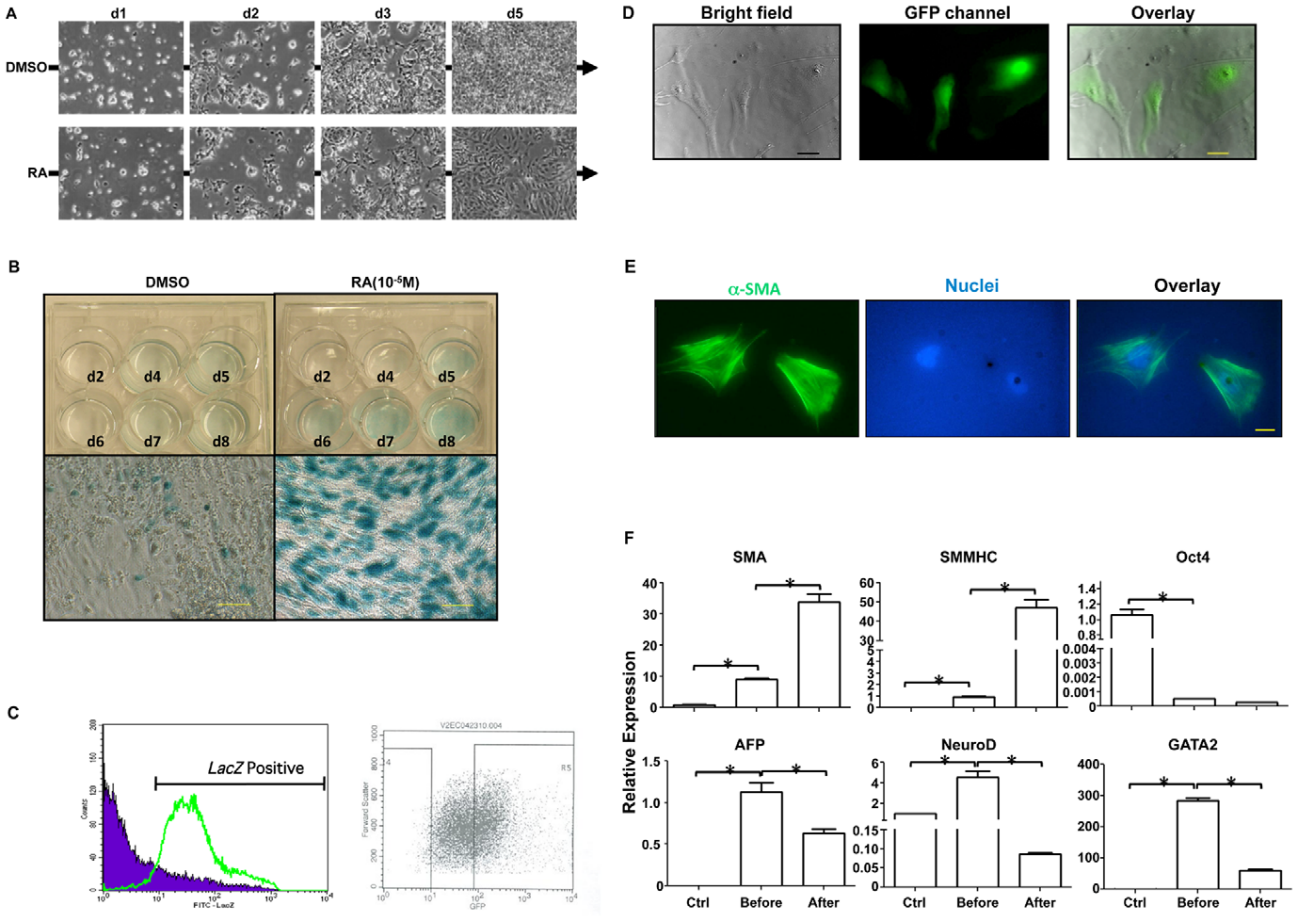
Sorting of SMCs from RA-induced SM22α^−/−^ ^***LacZ***^ **ESCs.** (A) Morphology change of SM22α^−/−LacZ^ ESCs under the treatment of DMSO (upper panel) or 10^−5^ M RA (lower panel) respectively as indicated time. (B) Comparison of β-gal staining of cells treated with either DMSO (left panel) or 10^−5^ M RA (right panel). The upper panel showed that β-gal staining positive cells accumulated with RA treatment time, while only sporadic β-gal staining positive cells existed in DMSO-treated cell population. The lower panel showed representative magnified images by day 8. Scale bar = 100 µm. (C–D) Using 5-chloromethylfluorescein di-β-D-galactopyranoside (FDG) to react with intracellular glutathione. In *LacZ*-positive cells derived from RA-induced SM22α^−/−LacZ^ ESCs, the FDG–glutathione adduct was converted to a bright green fluorescent product and *LacZ*-positive cells were subsequently sorted through a GFP channel (C) and cultured (D). Left panel: bright field; middle panel: GFP channel; right panel: merge. Scale bar = 100 µm. (E) Immunofluorescence staining of SMCs derived from sorted *LacZ*-positive cells with SMC-specific marker α-SMA antibody. The nuclei were co-stained with DAPI. Scale bar = 100 µm. (F) Gene expression of sorted cells analyzed by quantitative real-time PCR, compared to undifferentiated ESCs (control, Ctrl) or cells before sorting. Before: before sorting; After: after sorting. *p<0.05.

Under the treatment of RA, the SM22α^−/−*LacZ*^ ESCs monolayer underwent dramatic morphological changes. Throughout this process, the cells kept proliferating and gradually grew to confluence. In the meantime, the cells acquired an SMC-like shape starting from day 2 (Fig. 1A). Gradually, the SMC-like cells dominated the culture from day 5 and became confluent with a significant morphological difference compared with the DMSO treated spontaneous differentiation culture, which showed more random cellular shapes. The β-gal staining result indicated that RA treatment induced SM22α^−/−*LacZ*^ ESCs to differentiate into SMCs in a time-dependent manner (Fig. 1B). The number of β-gal positive cells increased with inducing time. In contrast, only sporadic staining was observed in spontaneously differentiated culture. Moreover, β-gal activity was dramatically enhanced in RA-treated SM22α^−/−*LacZ*^ ESCs compared to DMSO-treated cells at day 8 (data not shown). The differentiated cells were then sorted. After 6 days of RA induction, the derived SMC population from SM22α^−/−*LacZ*^ ESCs were sorted by FACS with hypotonic introduction of 5-chloromethylfluorescein di-β-D-galactopyranoside (FDG), which is designed to react with intracellular glutathione and subsequent hydrolysis by β-galactosidase and generate fluorescent adduct [13]. To eliminate the possibility of contamination of non-specific cell types with low-level transient SM22α gene expression, the sorting gate threshold was set to a value higher than 10^2^ fluorescence intensity. As a result, the sorted cells for further studies accounted for 20–25% of total *LacZ* positive cell population (Fig. 1C). Sorted cells displayed typical SMC-like morphology with a strong fluorescence signal (Fig. 1D) and strong expression of α-SMA evidenced by immunofluorescence staining (Fig. 1E). Gene expression analyses confirmed that much higher levels of the SMC specific genes, including α-SMA and SMMHC, were expressed in the purified cell population after FACS and simultaneously the expression level of both pluripotent marker (Oct4) and other cell lineage markers, such as endoderm marker AFP, mesoderm marker GATA2 and ectoderm marker NeuroD, was dramatically reduced (Fig. 1F). Collectively these results demonstrated that the above described method can be used for the enrichment of SMCs from the mixture of differentiated ESCs and thus allow us to generate purified SMCs in large quantities for further vascular tissue engineering applications.

### *In vitro* culture of sorted SMCs on 3D NF scaffolds

**Figure 2:**
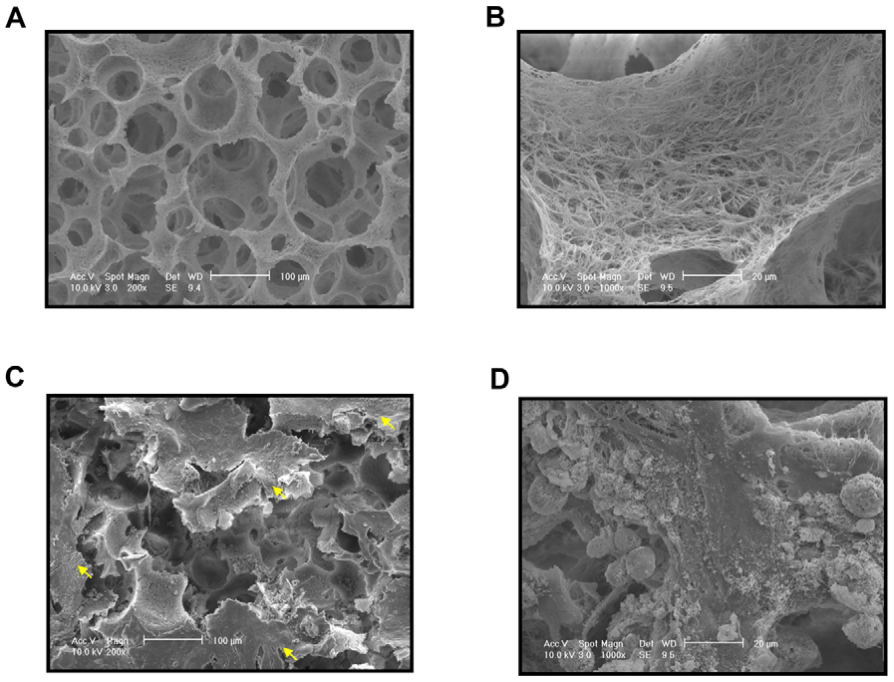
Representative SEM micrographs of NF scaffold and sorted SMCs seeded on the scaffold. (A–B) Low and high magnification images of the NF scaffold respectively. The cells were seeded and cultured for 24 hours and observed at low (C) or high (D) magnifications. Arrows indicate the cell aggregates inside the pores of scaffolds.

**Figure 3:**
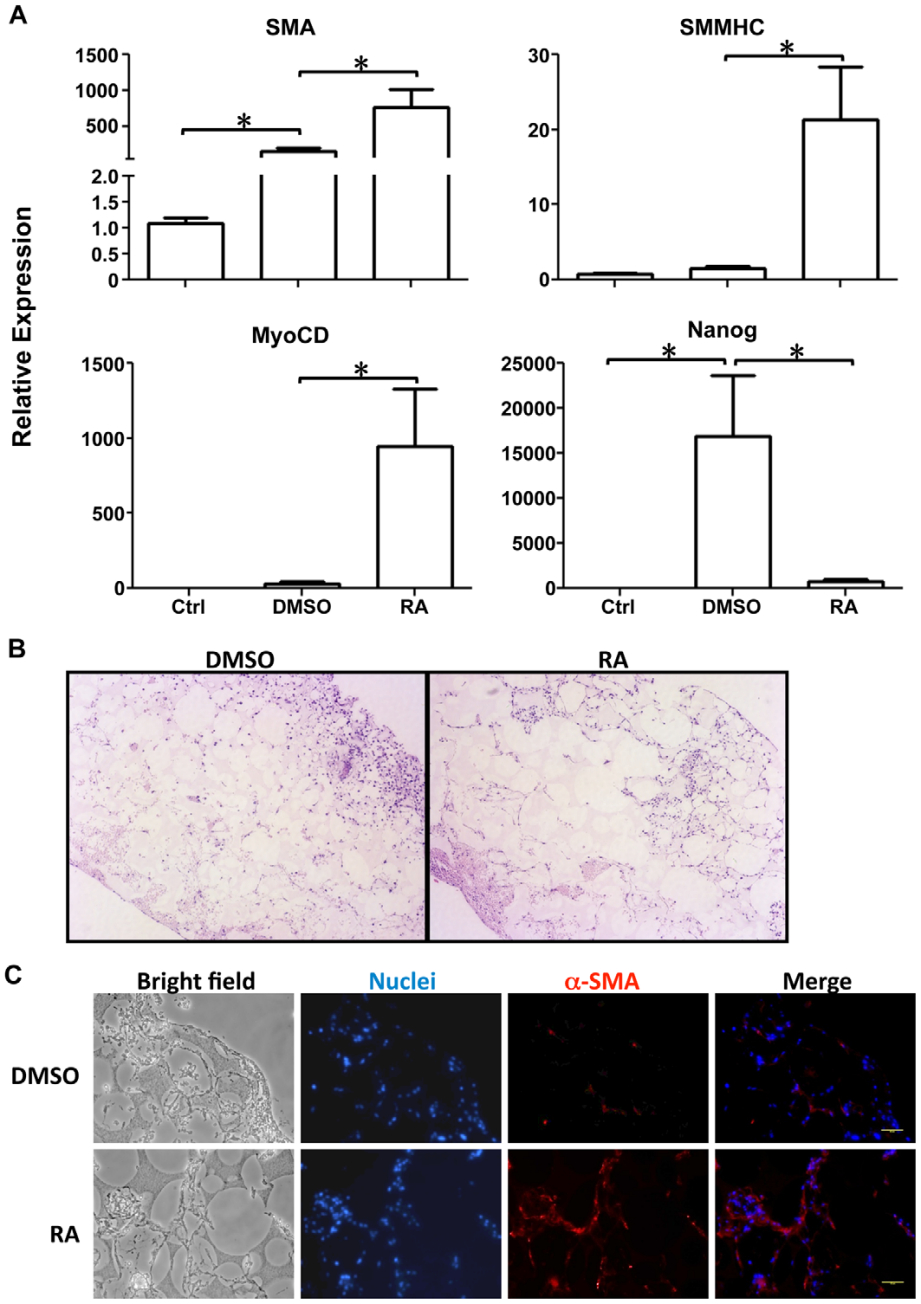
*In vitro* culture of sorted SMCs on 3D NF scaffolds. (A) Gene expression profiles of sorted SMCs before (Ctrl) and after seeded on NF scaffolds and cultured under the treatment of RA or DMSO for 2 weeks, respectively. (B) Histology of constructs cultured for 2 weeks with H–E staining. Left panel: DMSO treatment; right panel: RA treatment. (C) Immunofluorescence staining of α-SMA for constructs cultured for 2 weeks. Upper panel: DMSO treatment; Lower panel: RA treatment. Red: positive α-SMA expression; Blue: nuclei. Scale bar = 50 µm. *p<0.05.

Sorted SMCs were then seeded on 3D NF scaffolds (Fig. 2A&B) and cultured for 2 weeks in the medium containing DMSO or 10^−5^ M RA. After seeding for 24 hours, the cells aggregated inside the pores of the scaffolds (Fig. 2C&D). Gene expression analysis showed enhanced SMC marker gene expressions, including myocardin (MyoCD), α-SMA and SMMHC, for cells cultured on 3D NF scaffolds under continuous RA treatment, compared to DMSO treatment, while the expression of pluoripotent marker Nanog was kept inhibited by RA treatment (Fig. 3A). H–E staining showed that the cells distributed throughout the scaffold after 2 weeks of culture for both groups (Fig. 3B). However, immunofluorescence staining showed strong α-SMA positive staining in the constructs with continuous RA treatment. In contrast, weaker α-SMA staining was observed in the constructs treated with DMSO (Fig. 3C).

### Subcutaneous implantation of cell-scaffold constructs

**Figure 4:**
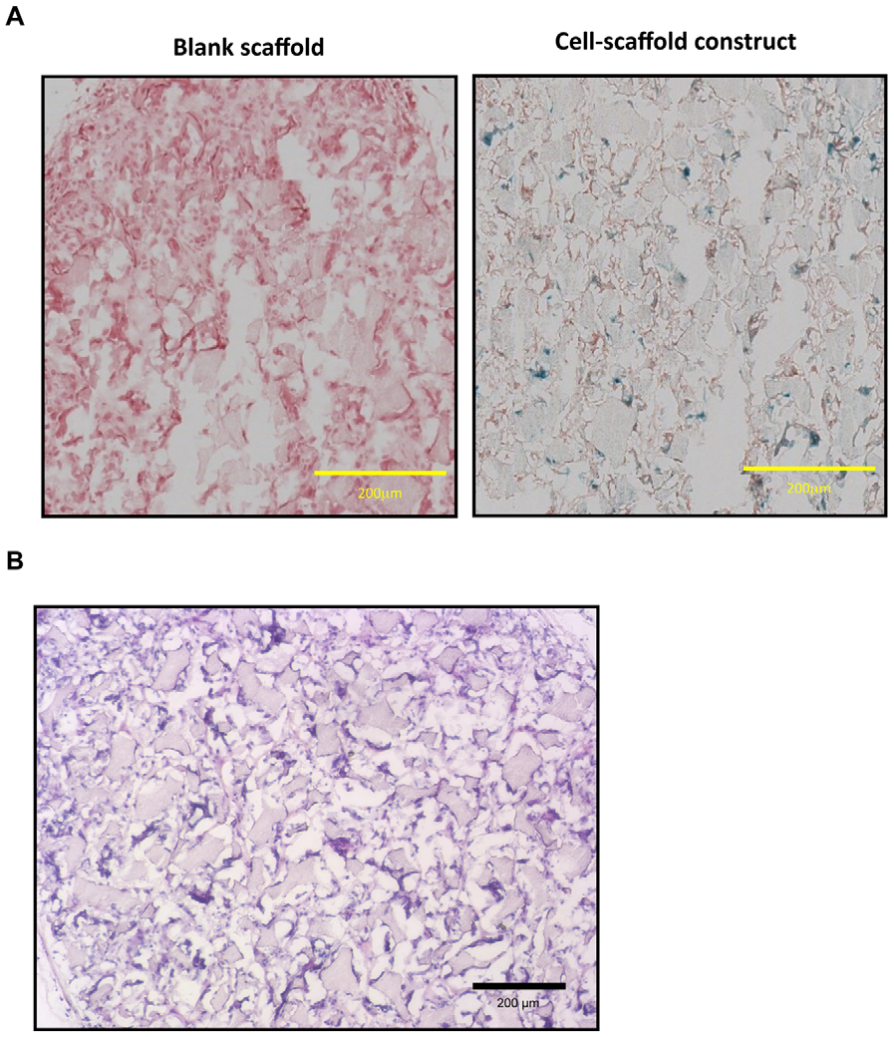
Histology of constructs implanted subcutaneously for 2 weeks. (A) β-gal staining. (Left) blank scaffold implants; (Right) cell-scaffold construct implants. Blue: positive *LacZ* staining; Red: nuclei. (B) H&E staining of cell-scaffold construct implants. Scale bar = 200 µm.

After the sorted SMCs were seeded on the scaffold for 24 hours, the cell-scaffold constructs were subcutaneously implanted into nude mice. After 2 weeks of implantation, the tissue infiltration into the scaffolds was observed on all samples. Positive *LacZ* staining of implanted cells was observed, while no detectable *LacZ* staining was observed in the blank scaffold implants (Fig. 4A), which indicated the survival of implanted SMCs inside the scaffold under *in vivo* conditions and retained the marker gene expression. Meanwhile, the teroatoma development was not observed on the cell-scaffold construct implants (Fig. 4B).

## Discussion

In this study, we presented a strategy to enrich SMCs from the differentiated ESCs and use the purified cells to construct vascular tissues on a macro-porous NF scaffold, which can serve as a potential platform for engineering therapeutic vascular tissues and for establishing vascular disease models. Although a number of *in vitro* SMC differentiation systems have been established, in which stem cells were induced to differentiate under the treatment of cytokines, growth factors or ECM components [8], [9], [15]–[19], there are limited publications showing the application of ESC-derived SMCs in vascular tissue engineering, largely owing to the heterogeneous properties of the cells. Given that ESCs have been considered to be a promising cell source for regenerating various tissues, we explored the capability of ESC-derived SMCs for constructing vascular tissues. SM22α is a 22 kDa protein that physically associates with cytoskeletal apparatus in contractile SMCs. SM22α-deficient (SM22α^−/−*LacZ*^) mice has been generated by gene targeting and used to study blood vessel development and gene function during embryogenesis, wherein, certain initiation codon of SM22α were replaced by the *LacZ* reporter [20], [21]. Previous study has shown that this replacement does not alter basal homeostatic function mediated by vascular SMCs in the developing mice and SM22α^−/−^ mice are viable and fertile [20]. Here, we used the ESCs, which have been employed for the generation of SM22α^−/−*LacZ*^ mice, to generate SMCs *in vitro* with *LacZ* staining as the positive selection marker. Naturally, the SM22α promoter is highly active in the heart tube and selectively expressed in a subset of arterial SMCs [20], [22]–[25]. Therefore, there was a possibility that cardiomyocytes might also be isolated using the SM22α promoter as the selecting marker. Our previous study has shown the SMCs, derived from ESCs or induced pluoripotent stem cells (iPSCs) by high dose (10^−5^ M) RA induction, had very low level of cardiomyocyte specific marker expression [8], therefore excluding the contamination of cardiomyocytes in our induction-sorting protocol. The results demonstrated that SM22α expression and the corresponding *LacZ* positive staining within the cell population sorted from differentiated ESCs exhibited a high degree of SMC specificity, as evidenced by the high level expression of SMC specific markers and diminished expression of other cell lineage markers.

A concern associated with the use of ESCs for therapies is the retention of undifferentiated pluripotent cells. Studies indicated that undifferentiated ESCs remained in embryoid bodies even after 28 days of differentiation and could be eradicated only by prolonged negative selection [18]. Undifferentiated ESCs are highly proliferative and have the potential to differentiate into unwanted cell lineages including teratoma formation, raising a significant concern over their use for therapeutic applications. The risk can be reduced if the ESCs are differentiated prior to transplantation [18], [26]. Here, we showed the absence of teratoma formation in our studies, likely benefited from the high efficiency of RA induced differentiation protocol and the positive sorting strategy. However, we observed that after being seeded and cultured on 3D NF scaffolds, the sorted cells could re-express the pluripotent genes (i.e., Nanog) when RA was withdrawn early from the culture medium, indicating that the sorted cells were not terminally differentiated and longer RA treatment may be necessary to drive the cells to a mature differentiated phenotype. Growth factors have also been shown to greatly affect the cell differentiation [27], [28]. The incorporation of a controlled-release capacity for RA or/and growth factors into the porous NF scaffolds [29] may further enhance functional vascular tissue regeneration.

## Supporting Information

Table S1 Primers Used for RT-PCR.
(DOC)
