# Supplementary material for: Construction of Vascular Tissues with Macro-Porous Nano-Fibrous Scaffolds and Smooth Muscle Cells Enriched from Differentiated Embryonic Stem Cells
Source: PLoS One. 2012 Apr 24;7(4):e35580. doi: 10.1371/journal.pone.0035580 (PMC3335865; doi:10.1371/journal.pone.0035580)
Supplement: Table S1 — Primers Used for RT-PCR. (DOC) [file pone.0035580.s001.doc]

| **Gene** | **Primer Sequence** | **Sequence ID** | **Product Size (bp)** |
| --- | --- | --- | --- |
| 18S RNA | forward: 5'-ggaagggcaccaccaggagt-3'  reverse: 5'-tgcagccccggacatctaag-3' | NR_003278 | 317 |
| MyoCD | forward: 5'-gtgggcccagcattttcaac-3'  reverse: 5'-tttccggtatcgtgctttcctc-3' | NM_145136 | 156 |
| α-SMA | forward: 5'-ggcatccacgaaaccacctat-3'  reverse: 5'-agccaccgatccagacagagta-3' | NM_007392 | 214 |
| SMMHC | forward: 5'-atgctgggaaggtggactacaa-3'  reverse: 5'-gtgcggaacatgcccttttt-3' | NM_013607 | 216 |
| OCT4 | Forward: 5’-gaaggtattcagccaaacga-3’  Reverse: 5’-aaattctccaggttgcctct-3’ | [NM_013633](http://www.ncbi.nlm.nih.gov/nuccore/NM_013633.2) | 216 |
| AFP | Forward: 5’-ggcctcttccagaaactagg-3’  Reverse: 5’-ccacaggccaatagtttgtc-3’ | [NM_007423](http://www.ncbi.nlm.nih.gov/nuccore/NM_007423.4) | 170 |
| GATA2 | Forward: 5’-agacgacaaccaccacctta-3’  Reverse: 5’-atgcactttgacagctcctc-3’ | [NM_008090](http://www.ncbi.nlm.nih.gov/nuccore/NM_008090.5) | 205 |
| NeuroD1 | Forward: 5’-ctttcaaacacgaaccatcc-3’  Reverse: 5’-aactgacgtgcctctaatcg-3’ | [NM_010894](http://www.ncbi.nlm.nih.gov/nuccore/NM_010894.2) | 237 |
| Nanog | forward: 5'- gatgcaagaactctcctcca-3'  reverse: 5'- caatggatgctgggatactc-3' | NM_028016 | 168 |
